# Increasing Rates of Infective Endocarditis in Patients with Inflammatory Bowel Disease

**DOI:** 10.7759/cureus.6919

**Published:** 2020-02-08

**Authors:** Sardar M Shah-Khan, Sardar M Shah-Khan, Fahad Alqahtani, Justin T Kupec

**Affiliations:** 1 Department of Internal Medicine, West Virginia University School of Medicine/Ruby Memorial Hospital, Morgantown, USA; 2 Department of Gastroenterology and Hepatology, West Virginia University School of Medicine/Ruby Memorial Hospital, Morgantown, USA; 3 Division of Cardiovascular Disease, West Virginia University School of Medicine/Ruby Memorial Hospital, Morgantown, USA

**Keywords:** infective endocarditis, inflammatory bowel disease, epidemiology

## Abstract

Introduction and aim

Infective endocarditis (IE) cases are on the rise in the United States. The incidence of IE in patients with inflammatory bowel disease (IBD) has not been reported. Utilizing a national level database, we sought to estimate the incidence of IE in IBD-related hospitalizations and to determine its outcomes.

Methods

Discharge records from the National Inpatient Sample were analyzed, and the International Classification of Diseases, ninth revision, Clinical Modification codes (ICD-9-CM) was used to identify adult patients with IBD (Crohn’s disease or ulcerative colitis) and IE between 2003 and 2014. Trends in the incidence of IE were recorded and multivariable regression was used to determine the impact of IE on IBD-hospitalizations.

Results

The incidence of IE in patients with IBD rose from 14.5 cases per 10,000 admissions in 2003 to 21.7 in 2014. After multivariable adjustment, both patient groups with CD (odds ratio [OR] 3.5, 95% confidence interval [CI] 3.0-4.1) and UC (OR 2.9, 95% CI 2.5-3.5) admitted with IE were found to be at greater risk for in-hospital mortality compared to non-IE admissions. Patients with IBD admitted with IE were found to have greater mean length of stay (13 days vs. six days, p<0.0001) and higher average hospital charges ($36,869.85 vs. $13,324.01, p <0.0001) compared to non-IE admissions.

Conclusions

Infective endocarditis is a growing complication in patients with IBD and is associated with increased mortality and utilization of healthcare resources. Further studies addressing the association between IE and IBD are needed.

## Introduction

Epidemiologic studies reveal that the incidence of infective endocarditis (IE) in the United States is increasing [1]. Between 2000 to 2011, hospital stays attributed to IE have been estimated to have increased by more than half [2]. Such a rise has been attributed to an increase in the number of at-risk populations for developing IE [1, 3-4]. Increased survival in IE-prone groups, such as those with prosthetic or implantable cardiac devices and those with congenital heart disease, is believed to play a role [5-7]. Furthermore, rising rates of intravenous drug use (IVDU) and a rise in the prevalence of immunosuppressed patients are likely to contribute [3-4, 7]. Altogether, IE represents a significant burden on the healthcare industry with higher rates of mortality and overall hospital costs compared to other hospitalizations [1, 8].

Infective endocarditis is a rare but reported extraintestinal complication of inflammatory bowel disease (IBD) [9-11]. Patients with IBD, potentially representing a high-risk group compared to the general population, are often immunosuppressed requiring multiple courses of corticosteroids and immunomodulatory therapies. Furthermore, these patients frequently undergo multiple procedures that render them susceptible to transient bacteremia and, subsequently, endocarditis.

Literature pertaining to IE in patients with IBD is limited to case reports and a single-center study reporting an association of IE with IBD [12]. As such, disease burden in this specifically vulnerable population may be underestimated. As more patients are diagnosed and treated for IBD, it is reasonable to hypothesize that rates of IE in these patients are increasing. This study sought to estimate the incidence of IE in IBD-related hospitalizations while also assessing the outcomes of IBD patients admitted with IE using a nationwide database.

## Materials and methods

Data source

A retrospective study was conducted using data from the Healthcare Cost and Utilization Project’s (HCUP) National Inpatient Sample (NIS) between the years 2003 and 2014. The NIS is sponsored by the Agency for Healthcare Research and Quality (AHRQ) and is the largest all-payer inpatient care database publicly available in the United States. It contains de-identified patient information pertaining to up to eight million discharges from approximately 1000 non-federal hospitals in 45 states. This data is stratified to represent approximately 20% of US inpatient hospitalizations across various hospitals and geographic regions. Estimates of national-level data were calculated using the sampling and weighting methods provided by the AHRQ.

Study cohort

**Figure 1:**
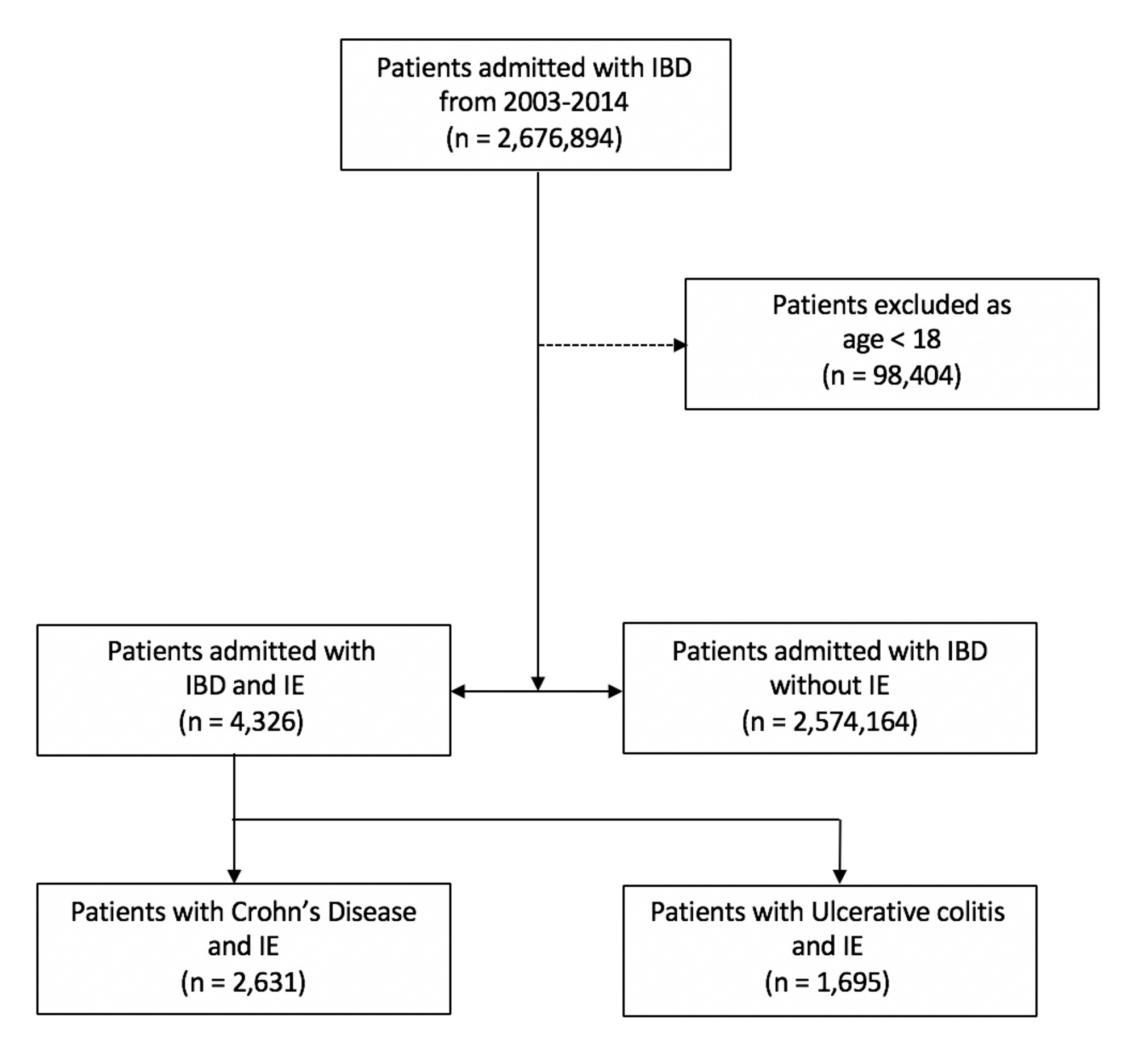
Flow chart demonstrating the patient sample population IBD - inflammatory bowel disease; IE - infective endocarditis

The International Classification of Diseases, ninth revision, Clinical Modification codes (ICD-9-CM) were used to identify adult patients discharged with a diagnosis of Crohn’s disease (CD) or ulcerative colitis (UC). All patients with IBD were evaluated for a concomitant diagnosis of infective endocarditis. Patients were divided into two groups: IBD without IE and IBD with IE (Figure 1). Patients within the IBD with IE group were thereafter divided into their respective groups of Crohn’s disease and ulcerative colitis. Baseline characteristics of both groups as well as additional demographic factors were characterized and documented as follows: race was classified as white, black, Hispanic, Asian/Pacific Islander, Native American or other, as characterized by the NIS; age, sex, primary payer source (Medicare, Medicaid, private, or other), hospital bed size (small, medium or large), hospital region (Northeast, Midwest, South, West), urban or rural location, hospital teaching status and median household income. 

Variables and outcomes

In order to best capture patients with IBD within the NIS, admissions with an ICD-9-CM diagnosis code of 555.x or 556.x were selected. Using appropriate diagnoses code for infective endocarditis, IBD admissions which included a diagnosis of IE were then identified. The microbiology of causative organisms was classified into four groups - Staphylococcus, Streptococcus, gram-negative and fungi. We investigated predictors of outcomes, including in-hospital mortality, acute myocardial infarction, stroke, acute renal failure, as well as the likelihood of non-home discharges using univariate and multivariate logistic regression models. The following variables were included in the logistic regression model: age, sex, race, primary payer status, hospital region, location of hospital (urban/rural), median household income quartile, and comorbidities listed in the Elixhauser comorbidity index. The Elixhauser comorbidity index is a well-validated aggregate of 30 comorbid conditions used to predict in-hospital mortality [13, 14]. Comorbidity software provided by HCUP was used in the analysis of this study.

Statistical analysis

Descriptive statistics for categorical variables were expressed as frequencies with percentages and as means with standard deviation (SD) for continuous variables. Baseline characteristics for each group were compared using the Pearson 2 test for categorical variables and an independent-sample t-test for continuous variables. The incidence of hospitalized cases of IE was expressed as cases per 10,000 IBD hospitalizations. Time trend analysis was performed using the Cochran Armitage trend test. Both univariate and multivariate logistic regression were used to control for potential confounding effects of the previously mentioned variables. Values were expressed as odds ratios (OR) with 95% confidence intervals (CI) to determine the hospital outcomes in those patients. Length of stay was expressed as mean with standard deviation. Total hospital charges were adjusted for inflation and expressed as means. A type I error rate of <0.05 was used as a cut off for statistical significance for all applicable tests. Data was analyzed using SPSS version 25 (IBM Corporation, Armonk, USA).

Ethical considerations

All information was obtained from the NIS, which is a deidentified patient database without protected health information. This study was declared exempt by the institutional review board of West Virginia University, USA.

## Results

Table 1 presents the characteristics of IBD patients hospitalized with and without infective endocarditis. Patients with IE were older on average (mean age 61 vs. 52, p<0.0001), predominantly white, female and with Crohn’s disease (60.8% vs. 32.0% with UC, p<0.0001). There was a large aggregation of patients in the southern region of the United States, in urban areas, and patients were more likely to be admitted to teaching hospitals. Compared to patients without IE, patients admitted with IE were more likely to have the comorbid valvular disease (39.9% to 2.8%, p<0.0001), renal failure (20.5% vs. 6.6%, p<0.0001), and congestive heart failure (14.2% vs. 5.0%, p<0.0001).

**Table 1:** Baseline characteristics of hospitalized IBD patients stratified by the presence of IE IBD - inflammatory bowel disease; IE - infective endocarditis; SD - standard deviation; HIV - human immunodeficiency virus; AIDS - acquired immunodeficiency syndrome

	IBD without IE (n = 2,574,174)	IBD with IE (n = 4,326)	p-value
Age (mean, SD)	52 (19)	61 (18)	
Age groups (%)			<0.0001
18-20 y	2.7	1.1	
21-40 y	28.8	14.3	
41-60 y	33.0	30.0	
>61 y	35.5	54.7	
IBD type (%)			<0.0001
Ulcerative colitis	36.5	39.2	
Crohn's disease	63.5	60.8	
Female (%)	57.4	52.4	<0.0001
Race (%)			<0.0001
White	81.2	81.5	
Black	9.9	10.5	
Hispanic	5.3	4.0	
Asian or Pacific Islander	1.0	1.1	
Native American	0.4	0.9	
Other	2.3	1.9	
Primary payer (%)			<0.0001
Medicare	37.8	57.7	
Medicaid	11.2	9.1	
Private	42.2	27.7	
Other	8.8	5.5	
Median zip code income (%)			<0.0001
First quartile	22.7	20.4	
Second quartile	24.5	27.7	
Third quartile	25.7	24.9	
Fourth quartile	27.1	27.0	
Elixhauser index (mean, SD)	1.74 (1.14)	2.56 (0.812)	
Elixhauser index (%)			<0.0001
0	19.2	4.1	
1	23.7	8.4	
2	21.2	15.4	
>3	35.9	72.2	
Hospital size (%)			0.262
Small	12.5	11.7	
Medium	25.4	25.6	
Large	62.1	62.7	
Hospital region (%)			0.112
Northeast	26.5	25.4	
Midwest	19.4	20.7	
South	36.9	36.5	
West	17.2	17.4	
Teaching hospital (%)	51.8	55.7	<0.0001
Rural (vs urban) location (%)	11.5	14.0	<0.0001
Renal failure (%)	6.6	20.5	<0.0001
Chronic pulmonary disease (%)	16.0	22.7	<0.0001
Pulmonary circulation disorder (%)	1.3	8.8	<0.0001
Peripheral vascular disease (%)	3.6	11.0	<0.0001
Diabetes (%)	14.0	21.6	<0.0001
Congestive heart failure (%)	5.0	14.2	<0.0001
Valvular disease (%)	2.8	39.9	<0.0001
Hypertension (%)	35.0	48.2	<0.0001
Liver disease (%)	3.5	6.3	<0.0001
Coagulopathy (%)	3.7	14.5	<0.0001
Depression (%)	13.5	14.5	0.051
Alcohol use (%)	2.5	2.5	0.983
Tobacco use (%)	14.3	9.3	<0.0001
Drug abuse (%)	4.8	7.5	<0.0001
Hepatitis C (%)	1.5	3.6	<0.0001
HIV and AIDS (%)	0.3	0.9	<0.0001

Prevalence of infective endocarditis amongst hospitalized patients with IBD

**Figure 2:**
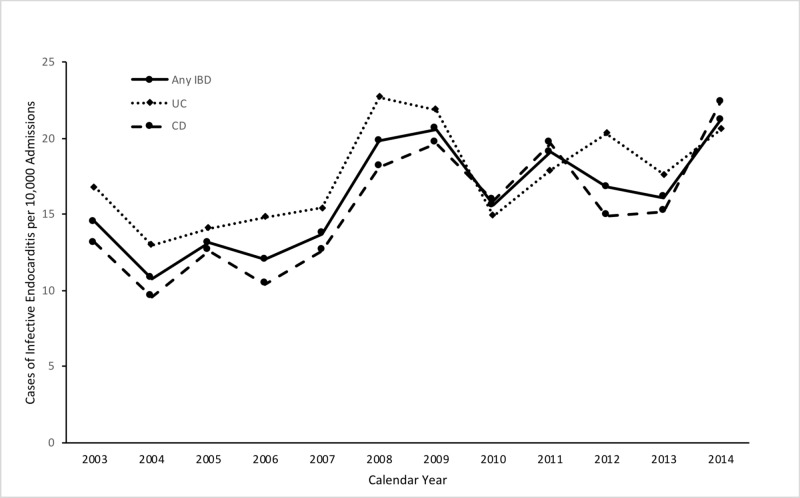
Graph demonstrating the incidence of infective endocarditis in patients with IBD from 2003-2014 IBD - inflammatory bowel disease; UC - Ulcerative colitis; CD - Crohn's disease

Between 2003-2014, there were 2,676,894 hospitalizations for patients with IBD. Among hospitalized patients with IBD from 2003-2014, there were a total of 4,326 with a discharge diagnosis of IE. Over this period, the rate of IE among patients with IBD rose from 14.5 cases per 10,000 admissions in 2003 to 21.7 cases in 2014 (Figure 2). In hospitalized patients with UC, the rate of IE increased from 16.8 cases per 10,000 admissions in 2003 to 20.6 in 2014 (pTrend <0.05). Among those hospitalized with Crohn’s disease, the absolute increase was greater with a rate of IE increasing from 13.1 cases per 10,000 admissions in 2003 to 22.4 (pTrend<0.05).

Association between in-hospital mortality and infective endocarditis

**Figure 3:**
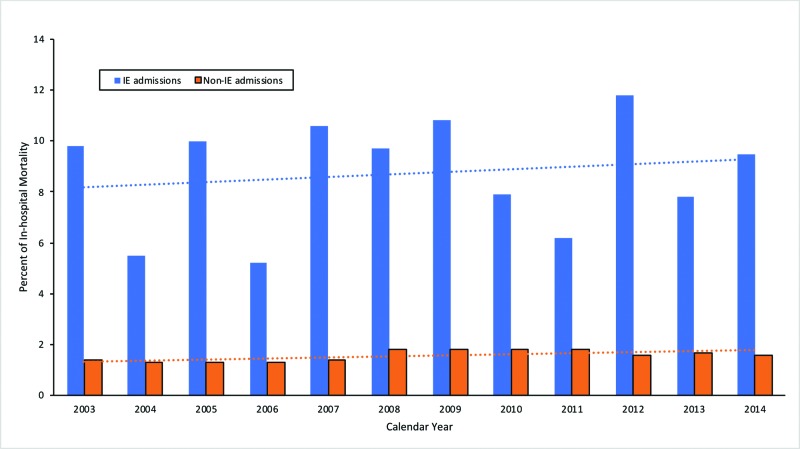
Graph demonstrating the trend of in-hospital mortality in IBD patients admitted with IE compared to non-IE hospitalizations IBD - inflammatory bowel disease; IE - infective endocarditis

Adjusting for age, sex, race, location, hospital characteristics, income, health insurance and comorbidities, multivariable logistic regression was used to assess the association between IE and the risk of in-hospital mortality (Tables 2 and 3). Among hospitalized patients with UC, patients with IE were found to have a strong association with in-hospital mortality (OR 2.9, 95% CI 2.5-3.5) compared to non-IE admissions. A similar but stronger association between IE and in-hospital mortality was found among patients with CD (OR 3.5, 95% CI 3.0-4.1). Overall, 9% of IBD patients admitted with IE died during their hospitalizations compared to 1.6% of non-IE admissions (Table 4). Figure 3 demonstrates the in-hospital mortality of IE admissions compared to non-IE admissions. While overall rates of in-hospital mortality have fluctuated, they favor an upward trend.

**Table 2:** Adjusted in-hospital outcomes for ulcerative colitis patients with infective endocarditis CI - confidence interval

		95% CI		
Outcome	OR	Lower	Upper	p-value
In-hospital mortality	2.9	2.5	3.5	0.0001
Acute myocardial infarction	2.9	2.3	3.6	0.0001
Stroke	3.8	3.1	4.6	0.0001
Acute renal failure	1.6	1.4	1.8	0.0001
Non-home discharge	3.6	3.2	4.0	0.0001

**Table 3:** Adjusted in-hospital outcomes for Crohn's disease patients with infective endocarditis CI: confidence interval

		95% CI		
Outcome	OR	Lower	Upper	p-value
In-hospital mortality	3.5	3.0	4.1	0.0001
Acute myocardial infarction	2.1	1.7	2.6	0.0001
Stroke	5.1	4.4	6.1	0.0001
Acute renal failure	1.8	1.6	2.0	0.0001
Non-home discharge	3.8	3.5	4.2	0.0001

**Table 4:** In-hospital outcomes of patients admitted with IBD stratified by presence of infective endocarditis IBD - inflammatory bowel disease; IE - infective endocarditis; MI - myocardial infarction

Outcome	IBD without IE	IBD with IE	p-value
Length of stay, days (25th, 75th percentile)	6 (2-7)	13 (4-17)	<0.0001
Mean cost, $ (25th, 75th percentile)	13,324.01 (4,383.52-13,411.38)	36,869.85 (9,167.93-42,168.62)	<0.0001
Acute MI (%)	1.1	4.2	<0.0001
Acute renal failure (%)	8.6	22.6	<0.0001
Stroke (%)	0.9	7.2	<0.0001
In-hospital mortality (%)	1.6	9.0	<0.0001
Non-home discharge (%)	26.7	68.2	<0.0001

Outcomes of hospitalized patients with IBD and infective endocarditis

Additional differences were observed in the outcomes in patients with inflammatory bowel disease admitted with IE. Patients admitted with IE were at greater risk and had a greater incidence of cardiovascular events, including acute myocardial infarction (MI), stroke, and acute renal failure (ARF) (Tables 2 and 3).

Outcomes related to the utilization of healthcare resources

**Figure 4:**
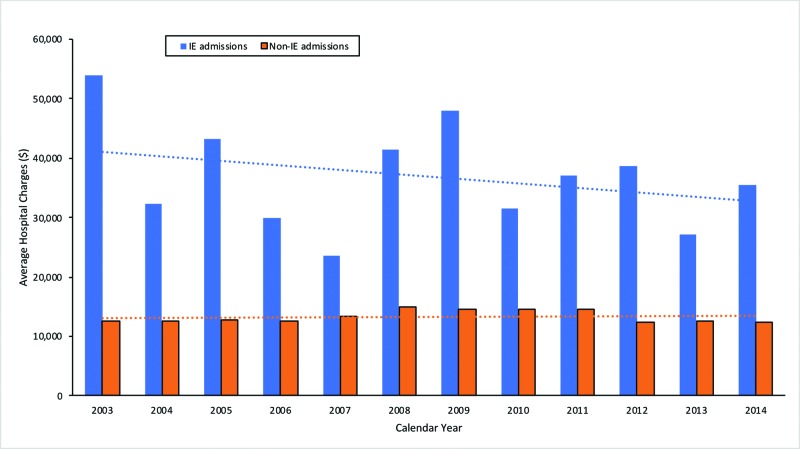
Graph demonstrating the trend in average hospital charges for IBD patients admitted with IE compared to non-IE hospitalizations IBD - inflammatory bowel disease; IE - infective endocarditis

A higher mean length of stay compared to non-IE admissions (13 days vs. 6 days, p <0.0001) was noted in patients with IE in the setting of IBD. Patients admitted with IE were more likely to have non-home discharges compared to non-IE admissions (UC [OR 3.6, 95% CI 3.2-4.0] and CD [OR 3.8, 95% CI 3.5-4.2]). After adjusting for inflation, admissions for IE were associated with significantly higher average hospital charges compared to non-IE admissions ($36,869.85 vs $13,324.01, p <0.0001) despite an overall downward trend in cost (Figure 4). Average hospital charges for IE admissions in 2014 were found to be significantly decreased compared to 2003 ($35,603 vs $53,948, p < 0.0001).

Organisms associated with infective endocarditis in patients with IBD

**Figure 5:**
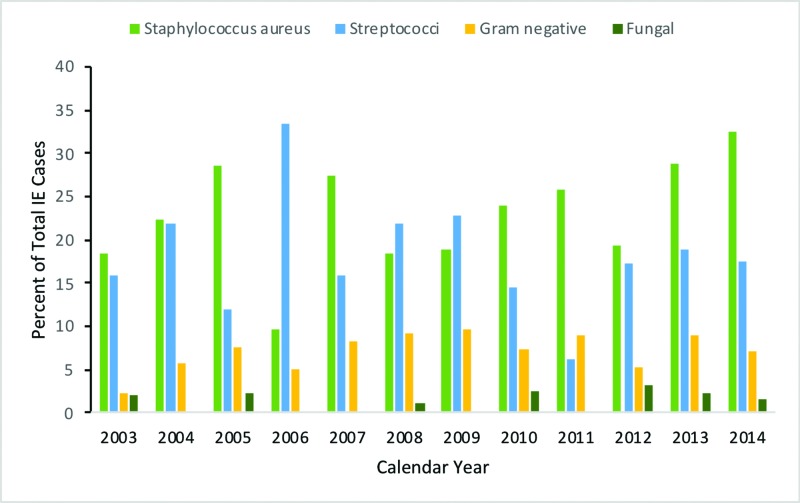
Graph demonstrating the trend in the microbiology associated with IE in patients with IBD IE - infective endocarditis; IBD - inflammatory bowel disease

Staphylococcus aureus was the most common causative organism of infectious endocarditis in IBD patients (Figure 5). Over the 11-year study period, the proportion of patients with Staphylococcus endocarditis increased from 18.4 % in 2003 to 32.5% in 2014 (p<0.001). Rates of Streptococcus species remained relatively stable from 16% in 2003 to 17.5% in 2014 (p<0.001). A significant rise in the proportion of patients with gram-negative species was seen, from 2.3% of isolates in 2003 to 7.1% in 2014 (p<0.001). Fungal IE decreased from 2.1% in 2003 to 1.6% in 2014 (p<0.001).

## Discussion

The burden of infective endocarditis in patients hospitalized with inflammatory bowel disease appears to be increasing and is more costly compared to non-IE hospitalizations. Additionally, patients with IBD hospitalized with IE suffer from higher rates of in-hospital mortality and a longer length-of-stay than non-IE hospitalizations. These findings parallel data reported on the general population [1, 2]. A rise in the number of at-risk populations, along with the increased rates of patient survival, is pushing the trend upward. Comorbid valvular disease (39% vs 2.8%), pre-hospitalization cardiac device placement (5.6% vs 0.1%) and comorbid renal failure (20.5% vs. 6.6%) were higher in hospitalized IBD patients with IE, consistent with existing data [2-4]. Surgical advances have allowed for increased valvular surgeries and a higher survival rate in patients with artificial valves. Similarly, the number of patients with chronic renal failure requiring dialysis and central venous catheters are on the rise [4]. These factors predispose patients to IE, but, interestingly, advances in echocardiography, and cardiac imaging may also contribute to increased diagnosis through improved recognition of the disease itself. While the opioid epidemic and IVDU have been reported to a contribute to the rise in the general population, its role in the rates of IE in patients with IBD is likely modest in comparison given the magnitude of the difference in comorbid drug abuse in patients with and without IE (7.5% vs. 4.8%, p<0.0001) [7].

An additional factor to consider is the paradigm shift in the treatment of IBD. Anti-tumor necrosis factor (TNF)-alpha antibody use has led to improvements in the rates of remission in patients with IBD [15-17]. Despite this, multiple studies have shown that these biologic therapies are not without adverse effects [18, 19]. Recent findings from the TREAT (Crohn’s Therapy Resource, Evaluation and Assessment Tool) registry demonstrated that patients with CD receiving infliximab had increasing rates of serious infection compared to patients on non-biologic therapies [19]. Similarly, a large retrospective study in France demonstrated that anti-TNF agents used in combination with thiopurines resulted in increased rates of opportunistic infections [20]. Growing rates of combination therapy and increasing the use of TNF-alpha agents may be a factor in the increasing rates of IE demonstrated in our findings. One limitation of the NIS is that its design does not allow us to assess the use of various drug therapies in our patient population. Thus, it is unable to ascertain from our study whether or not increasing the use of biologics plays a direct role in the increasing rates of IE.

Patients with inflammatory bowel disease with IE were shown to be at greater risk for in-hospital mortality compared to non-IE admissions. This is likely related to the increased incidence of adverse cardiovascular events in these patients as they were found to be at greater risk for acute MI, stroke, and acute renal failure. Additional factors to consider are independent risk factors for mortality in patients with IE. Studies of IE hospitalizations have demonstrated increasing age and infections due to Staphylococcus to be associated with higher rates of mortality [3, 8]. Given that a majority of IBD patients were older than 61 and cases of IE predominantly attributable to Staphylococcus, these likely contributed to the overall mortality rate in this population.

A number of factors likely account for the significantly increased lengths of stay and higher total hospitalization costs compared to non-IE hospitalizations. In addition to the significant morbidity these patients face, the treatment of IE requires up to six weeks of parenteral antibiotics and may even require surgery. Such a requirement causes these admissions to be inherently longer and thus more costly. Lengthy hospital stays resulting in debilitated patients only increases the likelihood that these patients will require further rehab on discharge. Furthermore, the need for parenteral antibiotics may represent an additional obstacle to home discharge, as those without the ability or support to administer the drugs are more likely to be discharged to skilled nursing facilities.

Due to the frequency with which patients with IBD undergo endoscopic procedures, it would make sense that the microbiology of IE would include more gram-negative organisms. A large retrospective study on the trend of IE in the general population showed that the proportion of IE due to gram-negative IE was approximately 8.4%, which is very similar in patients with IBD (7.6%) [2]. As noted above, Staphylococcus and Streptococcus species account for a majority of cases. Coupled together, these findings suggest that the source of IE in patients with IBD is likely similar to that of patients without IBD and is unrelated to the risks of bacteremia from endoscopy. Studies on the subsequent risk of developing IE in patients undergoing endoscopic procedures are limited. However, the present findings support previously published data that suggest there is no conclusive link between endoscopic procedures and the development of IE [21].

The American Society for Gastrointestinal Endoscopy (ASGE) guidelines on antibiotic prophylaxis for IE in patients undergoing endoscopy were introduced in 2015 and follow those set forth by the American Heart Association [21, 22]. In their update, antibiotic prophylaxis solely for the prevention of IE was no longer recommended. Prophylaxis was only recommended for patients with an established gastrointestinal infection and a cardiac condition in which IE represented a significant mortality risk: patients with prosthetic cardiac valves, patients with a history of IE, cardiac transplant patients with cardiac valvulopathy and patients with congenital heart disease. Furthermore, these guidelines only recommend prophylaxis with antibiotic agents directed against Enterococci as it is the abdominal organism most likely to cause IE.

The major strength of our study lies in its use of a large, nationwide database that assesses inpatient admissions. Given that the diagnosis of IE is almost always made on an inpatient basis, the NIS is uniquely equipped to capture a majority of these cases. The large sample size of the NIS provides our analysis with the power necessary to identify statistically significant findings in the trends and outcomes of IE in patients with IBD. Furthermore, the data provided in regard to demographics, hospital characteristics, and comorbidities allowed our analysis to control for potential confounders. The results of the present study add to the body of literature on opportunistic infections in patients with IBD.

Our study is not without limitations. Being a retrospective study, it is subject to the same inherent limitations that plague all such studies. Major caveats of the NIS should also be noted. Though the NIS allows for a large representation of national-level data, its accuracy relies heavily on the diagnostic coding of each patient’s conditions. While major diagnoses (i.e. infective endocarditis) most pertinent to a patient’s admission are unlikely to be missed, other predisposing (i.e. presence of a cardiac device) and comorbid factors (i.e. history of IVDU) that are less valuable to a hospital’s reimbursement are potentially undercoded. The design of the NIS does not allow us to identify cases of IE that were potentially missed during patient’s diagnoses, nor does it allow us to identify cases in those patients who never made it to admission. However, as noted previously, the diagnosis of IE is typically made on an inpatient basis making the former unlikely to significantly alter our findings. Conversely, the NIS treats each admission as a unique case. Patients with IE who were discharged and later readmitted, or those transferred from one facility to another, may falsely increase the number of cases of IE. 

## Conclusions

Infective endocarditis represents a growing complication in patients with inflammatory bowel disease. Compared to hospitalizations not related to IE, IBD patients with IE are at greater risk for adverse cardiovascular events and in-hospital mortality. Together these factors contribute to significant healthcare costs. While similar risk factors predispose patients with IBD to IE, and similar organisms are responsible relative to the general population, further studies are needed to assess this highly vulnerable population.
